# Birth weight, intrauterine growth restriction and nutritional status in childhood in relation to grip strength in adults: from the 1982 Pelotas (Brazil) birth cohort

**DOI:** 10.1016/j.nut.2015.08.014

**Published:** 2016-02

**Authors:** Renata Moraes Bielemann, Denise Petrucci Gigante, Bernardo Lessa Horta

**Affiliations:** a Postgraduate Program in Epidemiology, Federal University of Pelotas, Pelotas, Brazil; b Nutrition Department, Federal University of Pelotas, Pelotas, Brazil

**Keywords:** Cohort studies, Hand strength, Nutritional status, Birth weight

## Abstract

**Objective:**

The aim of this study was to evaluate the association among birth weight, intrauterine growth, and nutritional status in childhood with grip strength in young adults from the 1982 Pelotas (Brazil) birth cohort.

**Methods:**

In 1982, the hospital live births of Pelotas were followed. In 2012, grip strength was evaluated using a hand dynamometer and the best of the six measurements was used. Birth weight was analyzed as *z*-score for gestational age according to Williams (1982) curve. Weight-for-age, weight-for-length/height, and length/height-for-age at 2 and 4 y were analyzed in *z*-scores according to 2006 World Health Organization Child Growth Standards. Lean mass at 30 y was included as possible mediator using the g-computation formula.

**Results:**

In 2012, 3701 (68.1%) individuals were interviewed and 3470 were included in the present analyses. An increase of 1 *z*-score in birth weight was associated with an increase of 1.5 kg in grip strength in males (95% confidence interval, 1.1–1.9). Positive effect of birth weight on grip strength was found in females. Grip strength was greater in individuals who were born with appropriate size for gestational age and positively associated with weight- and length/height-for-age *z*-score at 2 and 4 y of age. A positive association between birth weight and grip strength was only partially mediated by adult lean mass (50% and 33% of total effect in males and females), whereas direct effect of weight at 2 y was found only in males.

**Conclusions:**

It is suggested that good nutrition in prenatal and early postnatal life has a positive influence on adult muscle strength. The results from birth weight were suggestive of fetal programming on grip strength measurement.

## Highlights

• Newborns from southern Brazil were followed for 30 y.
• Birth weight and nutritional status in childhood were prospectively measured.
• Grip strength was positively related to birth weight and early nutritional status.
• Coefficients of the association between birth weight and grip strength were higher in males.
• Birth weight was associated with adult grip strength independent of current lean mass.

## Introduction

Grip strength has been associated with several outcomes. In the clinical environment, low hand-grip strength is associated with postoperative complications, hospital length of stay, and short-term survival, whereas in epidemiological studies, grip strength is inversely related to all-cause mortality, disability, and risk for fracture but is positively related to functional status and bone mass [1].

While it has long been known that lifestyle conditions affect physical capacity as well as health status, the fetal origins of adult disease hypothesis, also known as the Barker hypothesis, provides additional insights based on a biological programming theory in which conditions in the womb determine susceptibility to diseases in later life [2]. Thus, considerable attention has been devoted to within-lifetime adaptation of the organism, termed *phenotypic plasticity* [3], which supports that experience in earlier life may predict adult health status. In particular, growth patterns in fetal life, infancy, and childhood have been associated with the components of the metabolic syndrome (hypertension, type 2 diabetes, and cardiovascular disease), implicating early nutrition as the underlying mechanism [4], [5], [6], [7], [8].

Evidence suggests that birth weight may be related to muscle strength in adulthood. Results from a meta-analysis observed a pooled effect of 0.86 kg (95% confidence interval [CI], 0.58–1.15) increase in muscle strength (mainly evaluated by grip strength) per additional kg of birth weight, which is maintained across the life span [9]. On the other hand, the effect of nutritional status in infancy and childhood has been scarcely evaluated, with studies suggesting that poor early growth is related to low muscle strength in children, adults, and the elderly [10], [11], [12]. Possible mechanisms involved in this long-term adaptation may include the number and type of muscle fibers [13], [14], [15], in addition to processes regulated by satellite cells [16].

Most of the studies on the long-term consequences of birth weight on muscular strength come from high-income countries [9]. However, investigations regarding this subject are important from low- and middle-income countries (LMICs) because there is uncertainty about the biological pathways that link low birth weight and stunting in childhood to later disease outcomes, as well as a possible difference in body composition at a given birth weight between high- and low-income countries [17]. Thus, establishing whether nutritional status during developmental periods is associated to outcomes related to adult noncommunicable diseases in LMICs in the same importance as in high-income countries is necessary [17].

Additionally, previous studies have not, to our knowledge, explored the mediating effect of the amount of lean mass in the relationship between early nutritional exposures and grip strength, especially regarding the use of adequate statistical tools. Recognizing these relationships may increase understanding of the underlying mechanisms that may explain the biological plausibility of this association. Moreover, the existing relationship between nutrition in infancy and childhood and later grip strength is not well established in the literature and this study may help in the construction of the body of evidence of studies analyzing the long-term consequences of early nutrition.

This study was aimed at assessing the role of birth weight, intrauterine growth restriction, and nutritional status in childhood on grip strength in young adults from the 1982 Pelotas (Brazil) birth cohort.

## Materials and methods

### Participants

In 1982, all maternity hospitals in the city were visited daily and the 5914 live births whose families lived in the urban area of the city were examined and their mothers interviewed. These individuals have been followed often throughout the years [8].

The study was approved by the Ethics Committee of the Medicine School of the Federal University of Pelotas. Written informed consent was obtained during the 2012–2013 follow-up before the interviews and physical evaluations.

### Infancy and childhood follow-ups

Maternity hospital staff used pediatric scales (Filizolla, Brazil; precision 10 g) to record birth weight. The scales were calibrated weekly. Gestational age was obtained by asking mothers the date of their last menstrual period. Term birth was defined as gestational age ≥37 wk. Information on prepregnancy weight was referred by the mother and was confirmed on the antenatal care register card. The research team measured maternal height soon after the women were admitted to the maternity hospital in 1982.

In 1984, a census was carried out in the urban area of the city in search of the cohort members; 4934 children were identified, which added to the 227 deaths representing a follow-up rate of 87.2%. Mean age at follow-up was 19.4 mo. In 1986, approximately 84.2% of the cohort children were located. In these visits, children were weighed using a portable spring scale with an accuracy of ±100 g (CMS Weighing Equipment, London, UK) and had their supine length (1984) or standing height (1986) measured using boards manufactured locally according to international specifications (AHRTAG, London, UK).

The weights in childhood were transformed to *z*-scores of weight, as well as the length/height at the 1984 and 1986 follow-ups, for age and sex using the 2006 World Health Organization growth standards [18]. Weight-for-length/height for sex was also transformed to *z*-score according to the same growth standard. Birth weight was transformed to *z*-score according to gestational age, using the reference population developed previously. Small-for-gestational-age was defined as a birth weight below the 10th percentile for gestational age and sex of the Williams' curve [19].

### Follow-up at 30 y

From June 2012 to February 2013, the cohort members were invited to visit the Epidemiologic Research Center, and 3701 individuals (follow-up rate: 68.1%, considering 325 known deaths) were examined and interviewed. Standing height was measured to the nearest 1 mm with barefooted participants using a wooden stadiometer. Weight was assessed using a BodPod^®^ scale with a precision of 0.01 kg. Body mass index (BMI) was calculated as weight in kg divided by the square of height in m.

Grip strength was obtained using the Jamar hand dynamometer (Lafayette Instrument Company, Lafayette, IN, USA). The assessment occurred with the participants in the sitting position in a chair with legs, back support, and fixed arms, with their shoulders adducted, their elbows flexed 90°, and their forearms in neutral, as recommended by the American Society of Hand therapists [20], and has high intratest and intertest reliability [21]. The hands in the device were positioned so that the thumbs were around one side of the handle and the four fingers were around the other side. Interviewees were able to hold the device comfortably in their hands and the position of the handle was altered if necessary. Participants were encouraged to squeeze as long and as tightly as possible or until the needle stopped. Three readings in total for each side were taken, alternately. The highest of the six grip strength measurements was considered in the statistical analysis. Exclusion criteria were pregnancy; tendinitis, current injuries or deterioration of mobility due to previous injury or accident in at least one of the arms or hands; fracture in the upper limbs in the previous 6 mo; wheelchair use; mental disorders; and degenerative diseases (e.g., fibromyalgia).

In 2012, lean mass was measured using the method of dual-energy x-ray absorptiometry (DXA; Lunar Prodigy Advance - GE^®^, Germany). Due to disparities between the sums of bone, fat, and lean mass measured by DXA and body weight measured by the previously described scale, the lean mass obtained by DXA was corrected according to body weight. Physical activity during commuting and leisure-time was measured in min/wk using the commuting section of the International Physical Activity Questionnaire and also a list of activities with frequency and duration of each activity, respectively.

### Statistical analyses

The analyses were performed using Stata 12 software (StataCorp, College Station, TX, USA). All analyses were stratified by sex and statistical interaction in the association between the exposures and grip strength according to sex was formally tested. The association between exposures and grip strength was evaluated using linear regression models. Wald test was used to test the significance and *P* = 0.05 was used to assign statistical significance. Maternal smoking during pregnancy, family income at birth, maternal prepregnancy BMI, maternal schooling, and skin color (evaluated by self-report in 2004–2005 and imputed maternal skin color for missing information) were considered possible confounders and the relationship of these variables with outcome was showed. Birth weight was used in the models that included nutritional status at the 1984 and 1986 follow-ups. Nutritional status at age 2 was included in the models using nutritional variables at the 1986 follow-up. A mediation analysis, using lean mass as a mediator, was carried out using the g-computation formula [22]. The formula is used to estimate the causal effect of time-varying exposures (early nutrition) on an outcome in the presence of later confounders (post-confounders) that are themselves affected by the exposures. It addresses the problem of estimating direct and indirect effects when the causal effect of the exposures on an outcome is mediated by intermediate variables (lean mass), and in particular when confounders (post-confounders) of the mediator–outcome relationships are themselves affected by the exposures. Thus, g-computation formula works with base confounders—variables that affect both main exposures and outcome—and post-confounders—variables not previously included in the model that are affected by exposures and related to the mediating variable. In this analysis, family income at birth, maternal schooling, maternal smoking during pregnancy, maternal prepregnancy BMI, skin color, birth weight, and nutritional status at age 2 were considered base confounders. Height and physical activity during commuting and leisure-time were considered post-confounders.

## Results

In the 2012–2013 visit, grip strength was measured in 3470 participants from the cohort (93.8% of the 3701 individuals interviewed). Percentage of data from males and females was similar. Characteristics of individuals with grip strength measurements are shown in Table 1. The prevalence of low birth weight was 6% and 9% for males and females, respectively (average birth weight for males was 3245.2 g, and for females, 3126.6 g). Most of the participants were white, from A/B economic level (highest), and had mothers with ≤8 y of study who did not smoke during pregnancy. More than half of the individuals were overweight and grip strength was higher in males (mean = 50.2 kg; SD = 8.2 kg) than in females (mean = 29.7 kg; SD = 5.4 kg).

Table 2 shows the average and prevalence of each exposure according to potential confounding variables, stratified by sex. The occurrence of the term birth was not related to any possible confounder. Family income at birth, maternal schooling, and maternal prepregnancy BMI were positively associated to birth weight *z*-score and nutritional status at 2 and 4 y in both males and females. Maternal smoking during pregnancy was associated with lower birth weight (*P* < 0.001), lower weight at age 2, and lower length for age *z*-score at 2 and 4 y in both sexes.

In males, maternal prepregnancy BMI was positively associated with adult grip strength (*P* = 0.005). Females whose mothers achieved ≥12 y of schooling had lower adult grip strength than their counterparts. Non-white females had ∼2 kg more strength than white females (*P* < 0.001; Table 3).

Table 4 shows that grip strength was not associated with gestational age in males. Males whose birth weight was appropriate for gestational age had on average 2.7 kg higher strength than males who were born small for gestational age. Men whose birth weight was >1.1 *z*-score had 8.5 kg (95% CI, 5.9–11.7) higher grip strength than males with birth weight <−2 *z*-score. Additionally, positive association was observed among males whose birth weight was between −2 and −1.1 *z*-score. Grip strength was also positively associated with all nutritional status variables at 2 y. Males with weight- and length-for-age and weight-for-length >1.1 *z*-score at 2 y had higher grip strength than males whose these nutritional status variables were <−2 *z*-score. Weight- and height-for-age *z*-score at 4 y were positively related to males' grip strength in young adulthood.

Table 5 shows that among females, birth weight was positively associated with adult grip strength (*P* < 0.001). Those females whose birth weight was appropriate for the gestational age had 1.2 kg higher strength than females who were born small for the gestational age. Regarding association between grip strength and nutritional status at 2 y, females whose weight-for-age was >1.1 *z*-scores had 2 kg (95% CI, 0.1–3.8) higher grip strength than females whose weight-for-age was more than 2 SD below the mean of the reference population. Weight-for-length *z*-score at 2 y was not associated with grip strength, whereas females with length-for-age >1.1 had higher grip strength than females with length-for-age <−2 z-scores. Similar to males, weight- and height-for-age *z*-score at 4 y were positively associated with adult grip strength in females. However, grip strength was positively associated with weight-for-length at 4 y (*P* = 0.019), since females with weight-for-height above p50 of the reference population had higher grip strength than females below the p50.

We observed an interaction between sex and most of the independent variables included in the analyses, the *P*-values for the variables weight-for-age *z*-score at 2 and 4 y, weight-for-length at 2 y, length- and height-for-age at 2 and 4 y were <0.001.

To test the mediation of lean mass, we also stratified the mediation analyses (Table 6). Results from weight-for-age and weight-for-height at 4 y in males and weight-for-length *z*-scores at 2 y in females were not showed in Table 6 because there was no total effect of these exposures on grip strength. All exposures showed a positive effect in grip strength at 30 y of age mediated by lean mass. Around 50% and 33% of total effect of birth weight on grip strength was explained by the mediator in males and females. Thus, the direct effect of birth weight was greater than the effect mediated by lean mass in females. No other variable had direct effect on adult grip strength in females. However, weight-for-age and weight-for-length *z*-scores at 2 y had a positive direct effect on grip strength in males. The percentage of effect on grip strength mediated by lean mass was 59% and 57% when considered weight-for-age and weight-for-length *z*-scores at 2 y, respectively.

## Discussion

Results of the present study demonstrated an important relationship between intrauterine growth and nutritional status at 2 and 4 y and grip strength in young adults belonging to the 1982 Pelotas birth cohort. Gestational age, however, was not associated with grip strength. The effect of most exposures was mainly mediated by lean mass in adulthood. However, birth weight according to the gestational age and weight-for-age *z*-score at 2 y in males had part of its effect not mediated by lean mass.

The main limitation of this study was the lack of data on birth length making it impossible to compare effects of birth weight and length. The present study, however, had the advantage of being population based and including several measurements at different ages. The high follow-up rate of the 1982 Pelotas birth cohort is another strength as it reduces the susceptibility to selection bias. Additionally, both exposures and outcomes were prospective and objectively measured.

A recent systematic review and meta-analysis found strong and consistent evidence for a positive association between birth weight and muscle strength in men and women across the life course [9]. Concerning evidence from LMICs, a study carried out in the Philippines showed a positive association between birth weight and adult grip strength only in males [23]. Birth weight was related to higher grip strength in Indian children, as well as conditional height and BMI from birth to 24 mo [10]. In Guatemala, intrauterine growth retardation was inversely related to grip strength in adolescence or in young adulthood [24].

The regression coefficients of the present study showed higher strength of association and more pronounced dose–response relationship in males. Other studies also found differences between sex in the relationship between early growth variables and later grip strength so that some associations were not statistically significant in females [11], [23], [25]. These disparities may be due to the different influence of sex hormones, such as testosterone [26], [27], which has anabolic effects on muscle and function [28], including strength in males [29]. It has been reported that among males, birth weight or weight gain in childhood are positively related to testosterone levels in adulthood [23], [30].

The association between birth weight and later grip strength can be explained by influences of birth size on growth and development of muscle fibers [31]. Although the concept that there is a fixed number of muscle fibers determined by birth, with subsequent growth only achieved by increasing fiber size, is probably outdated [31], results from the Hertfordshire Cohort Study found that muscle fiber score (fibers kg/mm^2^) from biopsy of the vastus lateralis was significantly lower in individuals with decreased birth weight in elderly males [32]. This finding lends support to the hypothesis that the number of muscle fibers is determined up to the birth, and subsequent growth is achieved by increasing size rather than the number of fibers [13].

The results indicated that both prenatal and postnatal period might play an important role on adult grip strength. A study in the United Kingdom found that grip strength in the elderly was positively associated with weight at 1 y [12]. On the other hand, a study carried out with participants from The Medical Research Council National Survey of Health and Development did not find association between weight and height at 2 y and adult grip strength, after adjustment for birth weight [33].

The theory of phenotypic plasticity explains lifetime adaptation of the organism due to early nutrition. In the case of muscle strength, the satellite cells, formed during myogenesis, play an essential role in the processes of postnatal muscle growth, muscle regeneration, and muscle hypertrophy across the life course. In animal models, the postnatal growth, a process that is normally regulated by satellite cells, is related to a phenotype marked by reduction in skeletal muscle mass and fiber caliber [16], which may help to explain the association found between nutritional status at 2 y and grip strength on later life. Additionally, hypertrophy is responsible for postnatal muscle growth, whereas hyperplasia occurs during prenatal period [15]. The hypertrophy helps to compensate for any deficit in fiber number, but it is suggested that muscle containing fewer fibers have larger type I fibers but not type II fibers [14]. This is important because the type II muscle fiber cross-sectional area seems to be associated with higher muscle strength [34].

In addition to the type of fiber, the amount of lean mass was important to mediate the association between early nutrition and later grip strength in our population. Several studies in diverse populations have supported the hypothesis that poor fetal growth, as measured by low birth weight, results in a smaller proportion of lean mass later in life [35], [36]. This increased susceptibility to lower values of lean mass and other negative outcomes (e.g., type 2 diabetes, hypertension, etc.) results from adaptations made by the fetus in an environment limited in its supply of nutrients, termed the *thrifty phenotype hypothesis*, which primarily reflects adaptation in growth of lean mass and competing organs [37].

The results pointed to no effect of gestational age on grip strength in adulthood, whereas birth weight according to gestational age, a proxy of intrauterine growth, was positively related to grip strength. It suggests that intrauterine growth is more important than duration of the gestation. Thus, more attention should be focused on the intrauterine environment, especially from 6 to 18 wk, when the primary muscle fibers are formed and after the formation of secondary fibers [38].

## Conclusions

The results of this study suggested that good nutrition in prenatal and early postnatal life positively influences adult muscle strength, evaluated in this study as grip strength. The results reinforce that the first 1000 d (conception to the first year of life) is a critical period for human growth and development. The findings indicate that the relationship between early determinants and adult grip strength may be mediated not only by muscle mass but by other causes not yet elucidated. The association with intrauterine growth emphasizes the importance of early environment and nutrition on later life. This is relevant because muscle strength is a marker of muscle function, which is related to loss of functional capacity, sarcopenia, and unfavorable cardiometabolic conditions in later life.

## Figures and Tables

**Table 1:** Characteristics of participants from the 1982 Pelotas (Brazil) birth cohort included in the analysis

Subjects' characteristics	Males		Females	
N	%	N	%
Maternal schooling (y)				
0–4	551	31.9	557	32.1
5–8	755	43.6	736	42.4
9–11	191	11.1	188	10.8
≥12	231	13.4	256	14.7
Maternal smoking during pregnancy				
Yes	597	34.5	608	35
No	1133	65.5	1131	65
Birth weight (g)				
<2500	109	6.3	158	9.1
≥2500	1621	93.7	1581	90.9
Skin color				
White	1341	75	1476	77.1
Non-white	446	25	438	22.9
Economic status at 30 y (ABEP∗)				
A/B (richest)	960	68.0	933	63.7
C	408	28.9	464	31.7
D/E (poorest)	44	3.1	67	4.6
Nutritional status at 30 y (BMI)				
Underweight (<18.5 kg/m^2^)	27	1.6	104	5.6
Normal (18.5–24.9 kg/m^2^)	625	35.6	809	43.7
Overweight (25–29.9 kg/m^2^)	714	40.7	515	27.8
Obese (>30 kg/m^2^)	388	22.1	425	22.9
Physical activity ≥150 min/wk				
Yes	1034	59.6	944	50.5
No	701	40.4	927	49.5
Grip strength (kg) [mean (SD)]	1730	50.2 (8.2)	1739	29.7 (5.4)
Lean mass (kg) [mean (SD)]	1596	56.7 (6.6)	1721	38.6 (5.3)

ABEP, Associação Brasileira de Empresas de Pesquisa; BMI, body mass index

∗ Criterion based on possession of certain consumer goods, head of the household's schooling, and presence of a maid.

**Table 2:** Description of main exposures according to socioeconomic and maternal characteristics of the 1982 Pelotas birth cohort

Subjects' characteristics	n	Birth weight *z*-score∗	Gestational age (≥37 wk)	Weight for age z score at 2 y	Weight for length *z*-score at 2 y	Length for age *z*-score at 2 y	Weight for age *z*-score at 4 y	Weight for height *z*-score at 4 y	Height for age *z*-score at 4 y
*P* value Mean (SD)	*P* value N (%)	*P* value Mean (SD)	*P* value Mean (SD)	*P* value Mean (SD)	*P* value Mean (SD)	*P* value Mean (SD)	*P* value Mean (SD)
Males									
Family income at birth (minimum wages)		<0.001	0.10	<0.001	<0.001	<0.001†	<0.001†	<0.001†	<0.001†
≤1	666	−0.51 (1.02)	387 (83.4)	−0.79 (1.16)	−0.06 (1.03)	−1.29 (1.18)	−0.40 (1.04)	0.52 (0.96)	−1.29 (1.18)
1.1–3	1463	−0.29 (1.01)	1001 (86.8)	−0.36 (1.12)	0.13 (1.03)	−0.79 (1.06)	−0.04 (1.02)	0.56 (1.02)	−0.70 (1.06)
3.1–6	544	−0.13 (1.05)	406 (86.4)	−0.04 (1.09)	0.26 (1.02)	−0.37 (1.06)	0.24 (1.04)	0.66 (1.08)	−0.35 (0.98)
>6	351	−0.01 (1.02)	281 (89.5)	0.31 (1.11)	0.48 (0.99)	−0.05 (1.02)	0.66 (1.18)	0.93 (1.20)	0.05 (0.96)
Maternal schooling (y)		<0.001†	0.080	<0.001†	<0.001	<0.001†	<0.001†	<0.001	<0.001†
0–4	1008	−0.36 (1.08)	630 (86.5)	−0.64 (1.18)	0.01 (1.05)	−1.11 (1.19)	−0.28 (1.06)	0.51 (1.00)	−1.06 (1.19)
5–8	1288	−0.31 (1.02)	879 (84.7)	−0.35 (1.11)	0.12 (1.02)	−0.75 (1.07)	−0.02 (1.01)	0.59 (1.02)	−0.71 (1.04)
9–11	330	−0.07 (0.98)	250 (89.9)	0.11 (1.07)	0.39 (0.97)	−0.28 (0.94)	0.37 (1.07)	0.70 (1.13)	−0.18 (0.93)
≥12	406	0.06 (0.98)	319 (88.4)	0.28 (1.08)	0.45 (1.00)	−0.06 (0.99)	0.59 (1.13)	0.85 (1.17)	0.03 (0.97)
Maternal smoking during pregnancy		<0.001	0.08	<0.001	0.71	<0.001	0.01†	0.14†	<0.001
No	1946	−0.15 (1.01)	1381 (87.3)	−0.23 (1.16)	0.17 (1.04)	−0.60 (1.12)	0.06 (1.06)	0.59 (1.01)	−0.58 (1.11)
Yes	1091	−0.49 (1.03)	699 (84.7)	−0.43 (1.18)	0.15 (1.02)	−0.94 (1.15)	−0.05 (1.14)	0.65 (1.12)	−0.83 (1.15)
Maternal prepregnancy BMI (kg/m^2^)		<0.001†	0.34	<0.001	<0.001	<0.001†	<0.001†	<0.001†	<0.001†
First quartile (≤20.1)	645	−0.48 (0.91)	432 (84.4)	−0.53 (1.09)	−0.06 (0.99)	−0.83 (1.08)	−0.27 (0.95)	0.26 (0.93)	−0.77 (1.02)
Second quartile (20.2–21.9)	650	−0.34 (0.97)	467 (87.0)	−0.32 (1.13)	0.12 (1.00)	−0.69 (1.19)	0.00 (1.04)	0.56 (0.96)	−0.64 (1.13)
Third quartile (22–24.6)	652	−0.24 (1.01)	467 (86.0)	−0.18 (1.14)	0.27 (1.02)	−0.65 (1.09)	0.18 (1.12)	0.76 (1.12)	−0.57 (1.11)
Fourth quartile (≥24.7)	619	0.03 (1.15)	440 (88.2)	−0.05 (1.18)	0.35 (1.05)	−0.53 (1.10)	0.26 (1.09)	0.82 (1.04)	−0.49 (1.12)
Females									
Family income at birth (minimum wages)		<0.001	0.50	<0.001	<0.001	<0.001	<0.001†	<0.001†	<0.001†
≤1	622	−0.53 (1.07)	358 (86.3)	−0.72 (1.13)	−0.02 (1.05)	−1.21 (1.14)	−0.48 (0.99)	0.33 (0.88)	−1.20 (1.12)
1.1–3	1325	−0.23 (1.03)	903 (86.3)	−0.29 (1.11)	0.17 (1.04)	−0.69 (1.07)	−0.10 (0.96)	0.47 (0.94)	−0.71 (1.02)
3.1–6	547	−0.21 (1.05)	420 (89.0)	−0.11 (1.19)	0.41 (1.11)	−0.31 (1.07)	0.35 (1.09)	0.75 (1.10)	−0.28 (1.08)
>6	366	0.07 (0.99)	280 (86.7)	0.30 (1.12)	0.46 (1.02)	−0.06 (1.12)	0.37 (0.97)	0.67 (0.96)	−0.14 (0.96)
Maternal schooling (y)		<0.001†	0.791	<0.001	<0.001	<0.001†	<0.001†	<0.001†	<0.001†
0–4	952	−0.33 (1.04)	561 (87.7)	−0.62 (1.15)	0.01 (1.04)	−1.07 (1.13)	−0.36 (1.02)	0.40 (0.94)	−1.08 (1.10)
5–8	1166	−0.28 (1.09)	818 (86.3)	−0.22 (1.10)	0.22 (1.04)	−0.64 (1.08)	−0.04 (0.95)	0.51 (0.89)	−0.66 (1.01)
9–11	324	−0.12 (1.01)	246 (88.2)	0.14 (1.22)	0.39 (1.13)	−0.26 (1.14)	0.31 (1.08)	0.64 (1.11)	−0.21 (1.00)
≥12	432	−0.09 (0.98)	345 (86.7)	0.31 (1.10)	0.47 (1.04)	−0.04 (0.99)	0.42 (1.02)	0.73 (1.10)	−0.12 (1.01)
Maternal smoking during pregnancy (y)		<0.001†	0.47	<0.001	0.16	<0.001†	<0.001†	0.85†	<0.001
No	1864	−0.10 (1.05)	1296 (86.6)	−0.15 (1.19)	0.23 (1.07)	−0.54 (1.16)	0.04 (1.06)	0.54 (1.00)	−0.56 (1.10)
Yes	1012	−0.53 (0.99)	674 (87.7)	−0.37 (1.14)	0.17 (1.04)	−0.83 (1.09)	−0.15 (0.98)	0.50 (0.91)	−0.84 (1.06)
Maternal prepregnancy BMI (kg/m^2^)		<0.001†	0.66	<0.001†	<0.001†	<0.001	<0.001†	<0.001†	0.003
First quartile (≤20.1)	595	−0.50 (0.97)	418 (86.5)	−0.51 (1.04)	−0.08 (0.94)	−0.76 (1.09)	−0.28 (0.92)	0.23 (0.86)	−0.76 (1.00)
Second quartile (20.2–21.9)	610	−0.35 (0.95)	424 (85.8)	−0.16 (1.13)	0.27 (1.07)	−0.58 (1.12)	−0.02 (0.98)	0.49 (0.88)	−0.59 (1.09)
Third quartile (22–24.6)	588	−0.19 (1.01)	441 (88.4)	−0.07 (1.18)	0.35 (1.06)	−0.54 (1.14)	0.06 (1.04)	0.57 (0.94)	−0.56 (1.11)
Fourth quartile (≥24.7)	616	0.12 (1.13)	446 (87.5)	0.04 (1.24)	0.41 (1.15)	−0.44 (1.05)	0.26 (1.11)	0.82 (1.07)	−0.51 (1.09)

BMI, body mass index

∗ According William's curve.

† Kruskal Wallis' test.

**Table 3:** Grip strength at age 30 y according to socioeconomic and maternal characteristics

Subjects' characteristics	Males			Females		
n	Mean (SD)	*P* value	n	Mean (SD)	*P* value
Family income at birth (minimum wages)			0.28			0.06
≤1	345	49.4 (8)		332	29.7 (5.5)	
1.1–3	842	50.4 (8.4)		867	30 (5.3)	
3.1–6	343	50.2 (7.7)		334	29.7 (5.6)	
>6	194	50.2 (8.2)		198	28.8 (5.3)	
Maternal schooling (y)			0.24			0.01
0–4	551	50.4 (8.1)		557	30.1 (5.5)	
5–8	755	49.8 (8.3)		736	29.9 (5.3)	
9–11	191	51 (8.4)		188	29.6 (5.1)	
≥12	231	50.1 (7.8)		256	28.8 (5.8)	
Maternal smoking during pregnancy			0.29			0.75
No	1133	50.0 (8)		1131	29.7 (5.5)	
Yes	597	50.4 (8.5)		608	29.8 (5.4)	
Maternal prepregnancy BMI (kg/m^2^)			0.005			0.65
First quartile (≤20.1)	360	49.5 (8.4)		359	29.4 (5)	
Second quartile (20.2–21.9)	384	49.1 (7.8)		368	30 (5.2)	
Third quartile (22–24.6)	381	50.9 (8.5)		360	29.5 (5.5)	
Fourth quartile (≥24.7)	368	50.8 (7.9)		390	30 (5.8)	
Skin color			0.19			<0.001
White	1296	50 (8.2)		1337	29.3 (5.2)	
Non-white	433	50.6 (8.2)		401	31.1 (5.6)	

BMI, body mass index

**Table 4:** Birth weight and growth at 2 y in relation to grip strength in males

Subjects' characteristics	n	Grip strength (kg)			
Crude		Adjusted	
β [95% CI]	*P* value	β [95% CI]	*P* value
Intrauterine growth retardation∗ (<10th percentile)			<0.001		<0.001
Yes	204	Reference		Reference	
No	1207	2.4 [1.2–3.6]		2.7 [1.4–4.0]	
Gestational age∗ (≥37 wk)			0.64		0.99
Yes	1240	0.31 [−1.0 to 1.62]		0.0 [−1.4 to 1.4]	
No	171	Reference		Reference	
Birth weight *z*-score∗ (according to William's curve)			<0.001		<0.001
<−2	55	Reference		Reference	
−2 to −1.1	255	3.6 [1.3–6]		3.6 [1.1–6.1]	
−1 to 0	510	4.6 [2.3–6.8]		5 [2.7–7.4]	
0.1–1	440	5.2 [3–7.5]		5.6 [3.2–8]	
>1.1	151	7.9 [5.4–10.4]		8.5 [5.9–11.2]	
Weight-for-age *z*-score at 2 y†			<0.001		<0.001
<−2	79	Reference		Reference	
−2 to −1.1	325	4.1 [2.1–6]		3.1 [0.5–5.7]	
−1 to 0	560	5.8 [3.9–7.7]		5.6 [3.1–8.2]	
0.1–1	402	8.2 [6.3–10.1]		7.5 [4.9–10.0]	
>1.1	213	9.1 [7–11.1]		8.9 [6.1–11.7]	
Weight-for-length *z*-score at 2 y†			<0.001		<0.001
<−2	17	Reference		Reference	
−2 to −1.1	166	4.9 [0.8–8.9]		5.4 [0–10.8]	
−1 to 0	517	6.5 [2.6–10.5]		6.5 [1.3–11.8]	
0.1–1	570	8.1 [4.2–12]		7.8 [2.5–13.1]	
>1.1	309	9.8 [5.9–13.8]		9.6 [4.3–15]	
Length-for-age *z*-score at 2 y†			<0.001		<0.001
<−2	175	Reference		Reference	
−2 to −1.1	411	2.7 [1.3–4.1]		2.8 [1–4.6]	
−1 to 0	586	4.3 [2.9–5.6]		4.5 [2.7–6.2]	
0.1–1	302	5.8 [4.3–7.3]		5.8 [3.9–7.8]	
>1.1	105	7.4 [5.4–9.3]		7.4 [4.9–9.8]	
Weight-for-age *z*-score at 4 y‡			<0.001		0.03
<−2	25	Reference		Reference	
−2 to −1.1	190	1.4 [−1.9 to 4.6]		−0.6 [−4.7 to 3.5]	
−1 to 0	558	4 [0.8–7.1]		1.1 [−3 to 5.1]	
0.1–1	528	6.8 [3.6–9.9]		2.6 [−1.7 to 6.9]	
>1.1	254	7.7 [4.5–11]		2.2 [−2.4 to 6.8]	
Weight-for-height *z*-score at 4 y‡			<0.001		0.55
<−1.1	82	−2.3 [−4.2 to −0.4]		−1.3 [−3.5 to 1]	
−1 to 0	323	Reference		Reference	
0.1−1	641	1 [−0.1 to 2.1]		−0.5 [−1.7 to 0.7]	
>1.1	507	2 [0.9–3.2]		−0.8 [−2.3 to 0.6]	
Height-for-age *z*-score at 4 y‡			<0.001		0.006
<−2	158	Reference		Reference	
−2 to −1.1	383	3 [1.6–4.5]		3.3 [1.3–5.3]	
−1 to 0	575	4.4 [3–5.8]		3.9 [1.7–6]	
0.1–1	324	6.7 [5.2–8.2]		4.7 [2.2–7.3]	
>1.1	113	7.5 [5.6–9.4]		5.5 [2.3–8.8]	

BMI, body mass index; CI, confidence interval

∗ Adjusted for family income at birth, maternal schooling, maternal smoking during pregnancy, maternal prepregnancy BMI, and skin color.

† Adjusted for family income at birth, maternal schooling, maternal smoking during pregnancy, maternal prepregnancy BMI, skin color, and birth weight.

‡ Adjusted for family income at birth, maternal schooling, maternal smoking at pregnancy, maternal prepregnancy BMI, skin color, birth weight, and nutritional status at 2 y.

**Table 5:** Birth weight and growth at 2 y in relation to grip strength in females

Subjects' characteristics	n	Grip strength (kg)			
Crude		Adjusted	
β [95% CI]	*P* value	β [95% CI]	*P* value
Intrauterine growth retardation∗ (<10th percentile)			0.02		0.008
Yes	192	Reference		Reference	
No	1195	1.01 [0.2–1.8]		1.2 [0.3–2.1]	
Gestational age∗ (≥37 wk)			0.41		0.74
Yes	1205	−0.4 [−1.2 to 0.5]		−0.15 [−1 to 0.7]	
No	183	Reference		Reference	
Birth weight *z*-score∗ (according to William's curve)			0.003		<0.001
<−2	53	Reference		Reference	
−2 to −1.1	250	−0.2 [−1.8 to 1.42]		−0.4 [−2.1 to 1.2]	
−1 to 0	522	0.3 [−1.3 to 1.8]		0.3 [−1.2 to 1.9]	
0.1–1	403	0.6 [−0.9 to 2.2]		0.9 [−0.7 to 2.5]	
>1.1	159	2 [0.3–3.6]		2.1 [0.4–3.9]	
Weight-for-age *z*-score at age 2 y†			<0.001		0.001
<−2	77	Reference		Reference	
−2 to −1.1	321	0.1 [−1.2 to 1.4]		−0.3 [−2 to 1.4]	
−1 to 0	588	1.1 [−0.1 to 2.4]		1.0 [−0.6 to 2.7]	
0.1–1	394	1.5 [0.2–2.9]		1.3 [−0.4 to 3]	
>1.1	220	2.1 [0.7–3.5]		2 [0.1–3.8]	
Weight-for-length *z*-score at age 2 y†			0.07		0.39
<−2	15	Reference		Reference	
−2 to −1.1	172	−2.7 [−5.5 to 0.2]		−2.2 [−5.6 to 1.2]	
−1 to 0	542	−2.4 [−5.1 to 0.4]		−1.7 [−5 to 1.6]	
0.1–1	533	−1.8 [−4.6 to 1.0]		−1.4 [−4.7 to 1.9]	
>1.1	337	−1.7 [−4.5 to 1.1]		−1.2 [−4.5 to 2.1]	
Length-for-age *z*-score at age 2 y†			<0.001		<0.001
<−2	174	Reference		Reference	
−2 to −1.1	402	0.2 [−0.8 to 1.1]		−0.4 [−1.5 to 0.8]	
−1 to 0	584	1.2 [0.3–2.1]		0.7 [−0.5 to 1.8]	
0.1–1	345	2.3 [1.3–3.3]		1.9 [0.7–3.1]	
>1.1	96	2.6 [1.3–3.9]		2.6 [1–4.3]	
Weight-for-age *z*-score at age 4 y‡			<0.001		<0.001
<−2	35	Reference		Reference	
−2 to −1.1	213	0.4 [−1.5 to 2.3]		1.0 [−1.5 to 3.6]	
−1 to 0	577	1.5 [−0.3 to 3.3]		2.7 [0.2–5.3]	
0.1–1	505	2.3 [0.5–4.2]		3.7 [1–6.4]	
>1.1	224	3.2 [1.3–5.1]		5.2 [2.3–8.1]	
Weight-for-height *z*-score at age 4 y‡			0.003		0.019
<−1.1	62	0.0 [−1.4 to 1.4]		0.0 [−1.6 to 1.6]	
−1 to 0	422	Reference		Reference	
0.1–1	647	0.7 [0.1–1.4]		0.9 [0.1–1.6]	
>1.1	422	1.4 [0.6–2.1]		1.5 [0.6–2.5]	
Height-for-age *z*-score at age 4 y‡			<0.001		0.008
<−2	166	Reference		Reference	
−2 to −1.1	419	1.3 [0.3–2.3]		1.9 [0.6–3.1]	
−1 to 0	554	1.7 [0.8–2.7]		1.5 [0.2–2.9]	
0.1–1	316	2.8 [1.8–3.8]		2.5 [0.9–4.2]	
>1.1	99	3.6 [2.3–4.9]		3.2 [1–5.3]	

BMI, body mass index; CI, confidence interval

∗ Adjusted for family income at birth, maternal schooling, maternal smoking during pregnancy, maternal prepregnancy BMI, skin color.

† Adjusted for family income at birth, maternal schooling, maternal smoking during pregnancy, maternal prepregnancy BMI, skin color, and birth weight.

‡ Adjusted for family income at birth, maternal schooling, maternal smoking during pregnancy, maternal prepregnancy BMI, skin color, and birth weight and nutritional status at 2 y.

**Table 6:** Total, direct, and indirect effects of infancy and childhood exposures on adult grip strength∗

Nutritional characteristics	Total association		Direct association		Indirect association	
β [95% CI]	*P* value	β [95% CI]	*P* value	β [95% CI]	*P* value
Males						
Intrauterine growth retardation (<10th percentile)	2.57 [1.32–3.81]	<0.001	0.92 [−0.25 to 2.10]	0.13	1.65 [0.95–2.34]	<0.001
Birth weight z score (according to William's curve)	1.48 [0.99–1.96]	<0.001	0.72 [0.21–1.23]	0.006	0.76 [0.41–1.10]	<0.001
Weight-for-age *z*-score at age 2 y	1.97 [1.44–2.51]	<0.001	0.80 [0.23–1.38]	0.006	1.17 [0.79–1.55]	<0.001
Weight-for-length *z*-score at age 2 y	1.4 [0.9–2]	<0.001	0.6 [0–1.2]	0.04	0.8 [0.5–1.2]	<0.001
Length-for-age *z*-score at age 2 y	1.67 [1.15–2.18]	<0.001	0.36 [−0.21 to 0.92]	0.21	1.31 [0.92–1.70]	<0.001
Height-for-age *z*-score at age 4 y	0.96 [0.20–1.73]	0.01	0.10 [−0.68 to 0.88]	0.80	0.86 [0.44–1.29]	<0.001
Females						
Intrauterine growth retardation (<10th percentile)	0.99 [0.12–1.86]	0.03	0.54 [−0.26 to 1.34]	0.18	0.45 [0.13–0.77]	0.006
Birth weight *z*-score (according to William's curve)	0.58 [0.26–0.90]	<0.001	0.39 [0.07–0.71]	0.02	0.19 [0.03–0.36]	0.02
Weight-for-age *z*-score at age 2 y	0.60 [0.28–0.92]	<0.001	0.06 [−0.28 to 0.41]	0.72	0.54 [0.33–0.75]	<0.001
Length-for-age *z*-score at age 2 y	0.83 [0.50–1.16]	<0.001	0.18 [−0.18 to 0.54]	0.33	0.66 [0.44–0.87]	<0.001
Weight-for-age *z*-score at age 4 y	1.27 [0.74–1.81]	<0.001	0.49 [−0.06 to 1.04]	0.08	0.78 [0.51–1.05]	<0.001
Weight-for-height *z*-score at age 4 y	0.5 [0.1–0.9]	0.03	0.1 [−0.4 to 0.5]	0.82	0.4 [0.2–0.6]	<0.001
Height-for-age *z*-score at age 4 y	0.58 [0.10–1.06]	0.02	0.19 [−0.30 to 0.68]	0.44	0.39 [0.17–0.60]	0.001

BMI, body mass index; CI, confidence interval

∗ Variables included in the analysis as base confounders were family income at birth, maternal schooling, maternal smoking during pregnancy, maternal prepregnancy BMI, skin color, birth weight, and nutritional status at 2 y. Variables included in the analysis as post-confounders were height and physical activity during commuting and leisure-time. Mediator was lean mass.
